# Investigating the Role of Brain Natriuretic Peptide (BNP) and N-Terminal-proBNP in Thrombosis and Acute Ischemic Stroke Etiology

**DOI:** 10.3390/ijms25052999

**Published:** 2024-03-05

**Authors:** Rosanna Rossi, Duaa Jabrah, Andrew Douglas, James Prendergast, Abhay Pandit, Michael Gilvarry, Ray McCarthy, Petra Redfors, Annika Nordanstig, Turgut Tatlisumak, Erik Ceder, Dennis Dunker, Jeanette Carlqvist, István Szikora, Georgios Tsivgoulis, Klearchos Psychogios, John Thornton, Alexandros Rentzos, Katarina Jood, Jesus Juega, Karen M. Doyle

**Affiliations:** 1 Department of Physiology and Galway Neuroscience Centre, School of Medicine, University of Galway, University Road, H91 TK33 Galway, Ireland; d.jabrah1@universityofgalway.ie (D.J.); andrew.douglas@universityofgalway.ie (A.D.); prendej1@tcd.ie (J.P.); 2 CÚRAM–SFI Research Centre in Medical Devices, University of Galway, H91 W2TY Galway, Ireland; abhay.pandit@universityofgalway.ie; 3 Institute of Biotechnology and Biomedicine, Universitat Autonoma de Barcelona (UAB), 08193 Bellaterra, Spain; 4 Cerenovus, Block 3, Corporate House, Ballybrit Business Park, H91 K5YD Galway, Ireland; mgilvarr@its.jnj.com (M.G.); rmccart9@its.jnj.com (R.M.); 5 Department of Neurology, Sahlgrenska University Hospital, 41345 Gothenburg, Sweden; petra.redfors@gu.se (P.R.); annika.nordanstig@vgregion.se (A.N.); turgut.tatlisumak@gmail.com (T.T.); katarina.jood@neuro.gu.se (K.J.); 6 Department of Clinical Neuroscience, Institute of Neuroscience and Physiology, Sahlgrenska Academy at University of Gothenburg, 41345 Gothenburg, Sweden; 7 Department of Interventional and Diagnostic Neuroradiology, Sahlgrenska University Hospital, 41345 Gothenburg, Sweden; erik.ceder@vgregion.se (E.C.); dennis.dunker@vgregion.se (D.D.); jeanette.carlqvist@vgregion.se (J.C.); alexandros.rentzos@vgregion.se (A.R.); 8 Department of Neurointerventions, National Institute of Clinical Neurosciences, 1145 Budapest, Hungary; h13424szi@ella.hu; 9 Second Department of Neurology, “Attikon” University Hospital, National & Kapodistrian University of Athens, 157 72 Athens, Greece; tsivgoulisgiorg@yahoo.gr; 10 Stroke Unit, Metropolitan Hospital, 185 47 Piraeus, Greece; apsychoyio@yahoo.gr; 11 Department of Radiology, Beaumont Hospital, Royal College of Surgeons in Ireland, D02 YN77 Dublin, Ireland; johnthornton@beaumont.ie; 12 Neurology Department, Val d’Hebron Hospital, 08035 Barcelona, Spain; jesusmjuega@gmail.com

**Keywords:** brain natriuretic peptide, BNP, NT-proBNP, stroke biomarkers, thrombus, acute ischemic stroke, stroke etiology

## Abstract

The need for biomarkers for acute ischemic stroke (AIS) to understand the mechanisms implicated in pathological clot formation is critical. The levels of the brain natriuretic peptides known as brain natriuretic peptide (BNP) and NT-proBNP have been shown to be increased in patients suffering from heart failure and other heart conditions. We measured their expression in AIS clots of cardioembolic (CE) and large artery atherosclerosis (LAA) etiology, evaluating their location inside the clots, aiming to uncover their possible role in thrombosis. We analyzed 80 thrombi from 80 AIS patients in the RESTORE registry of AIS clots, 40 of which were of CE and 40 of LAA etiology. The localization of BNP and NT-BNP, quantified using immunohistochemistry and immunofluorescence, in AIS-associated white blood cell subtypes was also investigated. We found a statistically significant positive correlation between BNP and NT-proBNP expression levels (Spearman’s rho = 0.668 *p* < 0.0001 *). We did not observe any statistically significant difference between LAA and CE clots in BNP expression (0.66 [0.13–3.54]% vs. 0.53 [0.14–3.07]%, *p* = 0.923) or in NT-proBNP expression (0.29 [0.11–0.58]% vs. 0.18 [0.05–0.51]%, *p* = 0.119), although there was a trend of higher NT-proBNP expression in the LAA clots. It was noticeable that BNP was distributed throughout the thrombus and especially within platelet-rich regions. However, NT-proBNP colocalized with neutrophils, macrophages, and T-lymphocytes, suggesting its association with the thrombo-inflammatory process.

## 1. Introduction

Investigating the mechanisms of pathological clot formation and the etiology of acute ischemic stroke (AIS) is crucial. There is still much to learn about the roles of thrombus components in thrombus formation and stability, although huge progress has been made in the last years [1,2,3,4]. The diagnosis of the correct stroke etiology is also extremely important, since it affects both prognosis [5,6] and outcomes [7], as well as the formulation of strategies for stroke treatment [8] and the prevention of stroke recurrence [9,10]. The diagnosis of stroke etiology commonly uses the Trial of ORG 10172 in Acute Stroke Treatment (TOAST) criteria, which are based on clinical features and patient medical history, including data collected by tests such as brain imaging (computed tomography, CT and magnetic resonance imaging, MRI), cardiac imaging, duplex imaging of extracranial arteries, arteriography, and laboratory assessments for a prothrombotic state. The two main determined etiologies in cases of large artery occlusion are large artery atherosclerosis (LAA) and cardioembolism (CE) [8]. For an estimated 15–40% of ischemic strokes [11,12,13], it is not possible to determine any etiology, even after an extensive clinical investigation [14]. Therefore, it is necessary to consider alternative ways for the determination of stroke etiology. In the last few years, there has been great interest and investment in looking for diagnostic biomarkers of stroke etiology [15,16,17].

The physiological roles of natriuretic peptides include the relaxation of the vasomotor tone, the inhibition of sympathetic activity, the reduction in cardiac preload, the increase in renal blood flow, and the increase in natriuresis and diuresis [18]. It was shown that the production of natriuretic peptides by cardiomyocytes is triggered by inflammation and hemodynamic stress, which are important pathophysiological contributors to atrial fibrillation and thrombus development in the left atrial appendage [19]. BNP is initially synthesized in cardiomyocytes as a reaction to stretch as pre-proBNP and is then cleaved into proBNP. When secreted, proBNP is split into BNP, which is biologically active, and the remaining N-terminal proBNP (NT-proBNP), which is thought to be biologically inactive [20,21]. Several studies showed that the serum and/or plasma levels of cardiac natriuretic peptides, such as BNP and NT-proBNP, are increased in patients with cardioembolic stroke [22,23,24]. Therefore, their investigation as possible biomarkers of cardioembolic stroke etiology is particularly interesting. There is also evidence that high plasma/serum levels of cardiac natriuretic peptides might be indicators of stroke recurrence [25] and stroke severity [26].

To explore the potential of BNP and NT-proBNP as useful ischemic stroke biomarkers, we investigated BNP and NT-proBNP expression in blood clots retrieved from 80 patients with AIS, comparing BNP and NT-proBNP expression in clots of LAA and CE etiology. Finally, to investigate a possible causative link with inflammation, we investigated their location within the AIS clots through colocalization studies, focusing on NT-proBNP which was expressed in nucleated cells.

## 2. Results

### 2.1. Characteristics of the Patients

In Table 1, the baseline patient characteristics are reported. We did not find any difference between the LAA and the CE cohorts in terms of admission NIHSS score (H1 = 0.53, *p* = 0.818), rtPA administration (*X*^1^ = 0.050, *p* = 0.823), or final mTICI score (H1 = 0.397, *p* = 0.529). The occluded vessel was significantly different in LAA and CE patients (H1 = 16.460, *p* < 0.0001 *). The main differences lay in the number of cases with M1 occlusions, which represented 70% and 32.5%, respectively, of CE and LAA patients, and in the number of tandem occlusions, which accounted for 2.5% of CE and 35% of LAA occlusions.

### 2.2. BNP and NT-proBNP Expression Levels in AIS Clots Are Positively Correlated, and the Expression of Each Molecule Is Similar in LAA and CE Clots

We found a statistically significant positive correlation between BNP and NT-proBNP expression (Spearman’s rho = 0.668 *p* < 0.0001 *). However, we did not find any significant correlation between BNP expression and NIHSS score at admission (Spearman’s rho = 0.114, *p* = 0.323), rtPA administration (Spearman’s rho = 0.141, *p* = 0.211), occlusion location (Spearman’s rho = −0.188, *p* = 0.095), and final mTICI score (Spearman’s rho = 0.126, *p* = 0.267). We found similar results for NT-proBNP, whose expression was not correlated with NIHSS score at admission (Spearman’s rho = 0.165, *p* = 0.152), rtPA administration (Spearman’s rho = 0.045, *p* = 0.692), occlusion location (Spearman’s Rho = −0.036, *p* = 0.754), and final mTICI score (Spearman’s rho = 0.097, *p* = 0.390).

**Figure 1:**
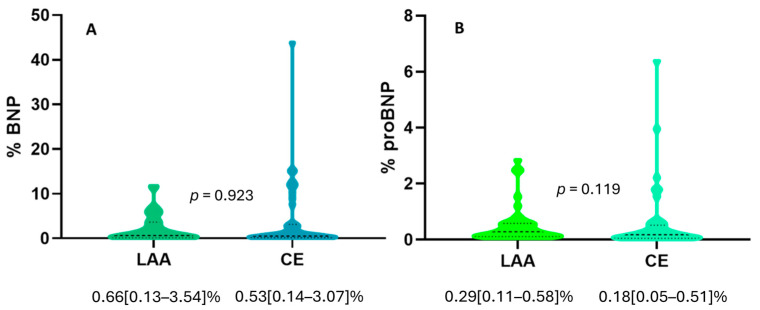
(**A**) Violin plot showing no difference in BNP expression in clots from patients with LAA (N = 40) or CE stroke etiology (N = 40). (**B**) Violin plot showing no difference in NT-proBNP expression in clots from patients with LAA (N = 40) or CE stroke etiology (N = 40). Dashed lines represent the median, while dotted lines represent the interquartile ranges, Q1 (lower dotted lines) and Q3 (upper dotted lines). The *p* value from Mann–Whitney U-test analysis is indicated.

Also, we did not observe any significant difference in BNP expression between LAA and CE clots (0.66 [0.13–3.54]% vs. 0.53 [0.14–3.07]%, *p* = 0.923), as shown in Figure 1A. Similarly, we did not find any significant difference in NT-proBNP expression between LAA and CE clots (0.29 [0.11–0.58]% vs. 0.18 [0.05–0.51]%, *p* = 0.119), although there was a trend of higher expression of NT-proBNP in LAA clots, as shown in Figure 1B.

**Figure 2:**
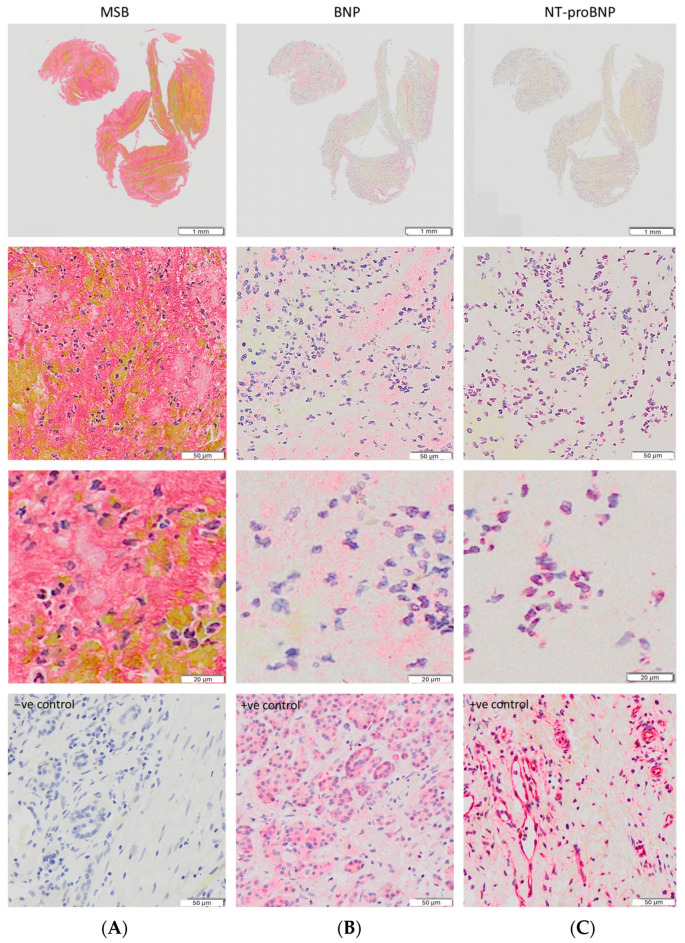
(**A**) MSB staining of the main clot components. (**B**,**C**) Expression of BNP and NT-proBNP in the same clot. BNP expression is located principally within platelet-rich regions, while NT-proBNP expression is only observed in nucleated cells. Higher magnification images are provided in the third row. In the fourth row, negative staining (**A**) and positive control staining images (pancreatic cancer tissue) for BNP (**B**) and proBNP (**C**) are shown. All images were captured using a 20× objective.

It was noticeable from the immunohistochemical staining (Figure 2) that while BNP was more diffuse in the clot and principally observed in platelet-rich regions, NT-proBNP expression colocalized with nucleated cells. This observation made us think that brain natriuretic peptides could be somehow involved in the mechanisms/pathways of pathological thrombosis, leading us to perform further colocalization experiments with markers of three WBC subtypes, i.e., CD3, a marker for T-lymphocytes, CD66b, a marker for neutrophils, and CD68, a marker for macrophages. In Supplementary Figure S1, IHC staining images of positive and negative controls for the examined white blood cell markers (CD3, CD66b, CD68, and a negative control) in human tonsils are provided.

### 2.3. NT-ProBNP Expression in AIS Clots Is Associated with T-Lymphocytes, Neutrophils, and Macrophages

**Figure 3:**
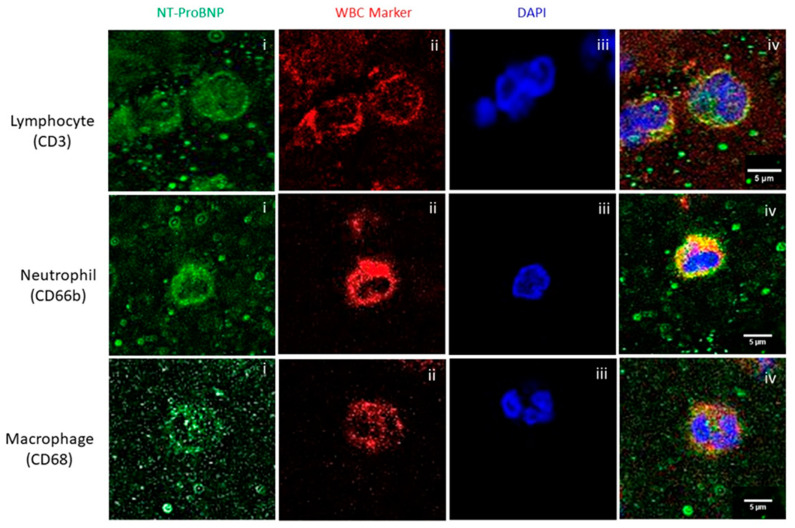
Colocalization analysis of NT-proBNP and WBC subtypes in AIS clots, showing individual signals (**i**–**iii**) and merged images (**iv**). Top row: T-lymphocytes (CD3); middle row: neutrophils (CD66b); bottom row: macrophages (CD68). All images were captured using a 60× objective (scale bar 5 μm).

Immunofluorescence staining revealed the association and colocalization of NT-proBNP with the three WBC subtypes we studied, i.e., T-lymphocytes (CD3), neutrophils (CD66b), and macrophages (CD68), as shown in Figure 3.

## 3. Discussion

Natriuretic peptides, including BNP and NT-proBNP, are biochemical markers included in international guidelines for the diagnosis and prognosis of heart failure [27]. Several studies showed that the serum and/or plasma levels of BNP and NT-proBNP are increased in patients with cardioembolic stroke [22,23,24], suggesting that they may have potential as possible biomarkers for stroke of CE etiology. We found no significant difference in the levels of either brain natriuretic peptide in CE and LAA AIS clots. However, BNP and NT-proBNP have also been proposed as biomarkers for atherosclerosis in acute coronary syndrome [28,29]. As coronary heart disease is the most common form of heart disease [30] and is of atherosclerotic origin, this may explain why we did not find any significant difference in BNP and NT-proBNP expression in AIS clots of LAA and CE etiology. In line with this, several recent studies associated high BNP/NT-pro BNP plasma levels with a higher risk of thrombotic events, although not only of cardioembolic etiology [31,32,33,34].

BNP and NT-proBNP are continuously released and are detectable in plasma. Their levels are increased upon ventricular myocardial wall stress, which is the main stimulus for their secretion [21]. As the plasma levels of NT-proBNP are higher than those of BNP, we expected that we might see higher expression of NT-proBNP than of BNP in AIS clots if their levels in clots are reflective of circulating proteins trapped within clots. However, we detected a 2–3-fold higher BNP expression than NT-proBNP expression. NT-proBNP expression in the AIS clots was predominantly located within nucleated cells. In contrast, BNP was principally distributed throughout platelet-rich regions of the thrombus, perhaps indicating a potential role in platelet activity. Interestingly, a correlation between BNP expression and mean platelet volume and a positive association of both features with heart failure have previously been shown [35]. This suggests that the bioactive BNP molecule may become trapped or stabilized within the clot structure perhaps through platelet association, while the biologically inert NT-proBNP molecule may not. Further research into the involvement of NT-proBNP and BNP in thrombosis is needed to explain this phenomenon.

The synthesis and secretion of natriuretic peptides can be induced by several circumstances, such as mechanical stress, systemic ischemia, hypoxia, and neurohumoral factors. Although the exact mechanisms of their regulation remain unclear, it is now generally accepted that mechanical stretch is the main cause of BNP expression rise in the myocardium [36]. However, several stimuli may act on BNP promoter elements through a variety of signaling pathways and affect their activity. For example, it was reported that activated T-lymphocytes produce inflammatory factors such as tumor necrosis factor, IL-1, and IL-6, which also selectively upregulate brain natriuretic peptide secretion [37]. This is in line with our findings that show colocalization between NT-proBNP and T-lymphocytes. Similarly, it is known that the recruitment of proinflammatory macrophages and granulocytes is one of the leading inflammatory processes causing cardiac depression and either new-onset heart failure or acute decompensation of chronic heart failure [38]. Further research is needed to understand the possible causative links with inflammation and the involvement of brain natriuretic peptides.

Our study has limitations. First, we acknowledge that our sample size was relatively small. Further studies including a higher number of patients would be useful to validate our findings. Furthermore, we only studied the LAA and CE etiologies. It would be useful to also study cryptogenic cases in future work. We acknowledge that the differences in occlusion location might represent a possible source of bias in our study, as there were significantly more M1 occlusions in the CE cases than in the LAA cases. Furthermore, we only included cases where all thrombus material was removed in the first pass, which may have introduced a bias. Although some studies showed that the BNP levels in the plasma of AIS patients correlated with stroke severity and outcome [26,39], we did not find any correlation between BNP or NT-proBNP levels and NIHSS score at admission or recanalization outcome (final mTICI score) in AIS clots. However, we acknowledge that we did not evaluate the correlation with other parameters of stroke severity and clinical outcome, such as cerebral infarct size, NIHSS score at discharge, or mRS scores, which were reported in a previous study [40]. It would also be useful to correlate the plasma BNP/NT-pro BNP levels with their expression levels in the clots in future studies. There is some evidence that the timing of BNP measurement is important, with the acute-phase levels being most indicative of the outcome. The BNP levels were shown to negatively correlate with a favorable outcome within 24 h of stroke onset [41]. Recent studies suggested that BNP and NT-proBNP may have use in predicting stroke risk in patients with heart failure with preserved ejection fraction [42], prognostic potential for functional outcome after AIS [43,44], and use in optimizing the management of stroke associated to heart disease [45]. Further work correlating BNP and NT-proBNP expression in AIS clots with longer-term patient outcome measures of stroke severity could yield further interesting insights. Future research investigating further the involvement of BNP and NT-proBNP in thrombo-inflammatory processes could lead to valuable advances in our understanding of the pathophysiology of ischemic stroke.

## 4. Materials and Methods

### 4.1. Patient Cohort

We selected 80 patients from the RESTORE registry of AIS clots, of which 40 patients with LAA stroke etiology, and 40 patients with CE stroke etiology. The RESTORE registry is a registry of thrombotic material extracted via mechanical thrombectomy from 1000 AIS patients during the period from February 2018 to December 2019 in four specialized stroke centers in Europe. This multi-center prospective study was performed in accordance with the ethical standards of the Declaration of Helsinki and its amendments [46] and was approved by the regional hospital ethics committees and the University of Galway research ethics committees (16-SEPT-08). We included only patients >18 years, having been treated with mechanical thrombectomy for AIS and whose thrombus material was available for analysis. Patients who recanalized without the need of performing mechanical thrombectomy or without thrombus material extracted were excluded from the Registry. For each patient, we collected an anonymized data abstraction form containing pertinent procedural data, such as previous rtPA administration, NIHSS score at admission, occlusion location, stroke etiology, and final mTICI score. Stroke etiology was reported according to the TOAST classification system [8]. The subset of 80 patients in this study were selected at random from cases of stroke with confirmed LAA or CE etiology, where the clot was removed in the first pass.

### 4.2. AIS Clot Collection and Processing

The blood clots extracted via mechanical thrombectomy were shipped to the University of Galway in pots containing 10% formalin. Upon arrival, a gross photo of each thrombus was taken with a Canon EOS 1300D camera (Canon Inc., Tokyo, Japan). Following this, the thrombi were placed in histological cassettes for tissue processing and paraffin embedding, maximizing longitudinal distal to proximal orientation, thereby ensuring that the tissue sections for analysis were representative of the whole clot length.

### 4.3. Immunohistochemistry Staining

We cut 3 µm sections from each paraffin-embedded block with a microtome, and BNP and NT-proBNP staining was performed by immunohistochemistry (IHC) on a Leica Bond-III autostainer using the BOND Polymer Refine Red Detection kit (Leica Biosystems, Wetzlar, Germany #DS9390). Antigen retrieval with tris-EDTA (Leica Biosystems, Wetzlar, Germany #AR9640) was performed for 20 min. the incubation time for the primary antibody rabbit anti-BNP (Abcam, Cambridge, UK, ab236101, 1:100 dilution) or rabbit anti-NT-proBNP (My BioSource Inc., San Diego, CA, USA, MBS2015959, 1:50 dilution) was 15 min, followed by 30 min of incubation with an anti-rabbit secondary antibody. Then, counterstaining of the tissue using hematoxylin was performed for 5 min. Finally, the sections were washed with a washing solution (Leica Biosystems, Wetzlar, Germany #AR9590), rinsed in distilled water, dehydrated in alcohol, cleared in xylene, and mounted with DPX. The negative controls were performed by omission of the primary antibody. Pancreatic cancer tissue (BioIVT, Westbury, NY, USA) was used as positive control tissue for BNP and NT-proBNP expression.

### 4.4. Slide Scanning and Quantification

The stained slides were scanned using an Olympus vs120 slide scanner at 20× magnification, generating digital whole slide scan images. The digital slides were then quantified using Orbit Image Analysis Software (www.orbit.bio), version 3.64 (9.6.2020) [47], as previously described [2]. In summary, we created two different models (exclusion and inclusion) to distinguish regions to be excluded (e.g., background and artefact) from regions containing the component of interest (BNP or NT-proBNP), enabling the molecules’ quantitative assessment.

### 4.5. Immunofluorescence Staining

Immunofluorescence staining was performed on a subset of samples in order to evaluate the colocalization of NT-proBNP with three WBC markers. We used the CD3 marker for T-lymphocytes, the CD68 marker for macrophages, and the CD66b marker for neutrophils. The primary antibodies used were as follows: rabbit anti-NT-proBNP (My BioSource, Inc., San Diego, CA, USA, MBS2015959, 1:50); mouse anti-CD3 (Abcam Cambridge, UK, ab17143, 1:10); mouse anti-CD68 (Abcam, Cambridge, UK, ab955, 1:50); and mouse anti-CD66b (Novus Biologicals, Centennial, CO, USA, NB100-77808, 1:100). Alexa Fluor 594-labelled goat anti-mouse IgG H&L (Abcam Cambridge, UK, ab150116, 1:200) and Alexa Fluor 488-labelled goat anti-rabbit IgG H&L (Abcam, Cambridge, UK ab150077, 1:200) were used as secondary antibodies.

Slides with 3 µm sections of thrombus tissue were deparaffinized with xylene, and the tissue was then rehydrated with serial 100%, 95%, 70%, and 50% *v*/*v* ethanol solutions. The slides were then incubated for 20 min in Tris-EDTA buffer in a microwave at 98 °C and washed with phosphate-buffered saline (PBS), followed by a wash with PBS containing 0.2% Tween 20 (PBS-Tx). The sections were then incubated with blocking buffer (3% normal goat serum, in PBS-Tx) for 1 h at room temperature under agitation. Incubation with two primary antibodies (anti-NT-proBNP and one of the anti-WBC primary antibodies per slide) followed at 37 °C for 1 h and then overnight at 4 °C. After washing, the sections were incubated with the secondary antibodies for 1 h at 37 °C and then coverslipped in the presence of the 4′,6-diamidino-2-phenylindole (DAPI) mounting medium for nucleic acid staining.

A FV3000 confocal laser scanning microscope (Olympus, Tokyo, Japan) was used to capture immunostaining images by using a 60× objective, and FIJI software (ImageJ, version 1.54h, 15 December 2023) was used to analyze the captured images.

### 4.6. Statistical Analysis

IBM SPSS-25 software was used for the statistical analyses, and GraphPad Prism 9.2.0 for graph creation. The quantitative variables did not follow a standard normal distribution, as indicated by the Kolmogorov–Smirnov test and the Shapiro–Wilk test. Therefore, the non-parametric Mann–Whitney U-test or the Kruskal–Wallis test with Dunn’s correction for multiple comparisons was used to assess the significance of the differences between the groups for the continuous variables. Chi-square analysis was used to assess the significance of the differences between the groups for the nominal variables. The non-parametric Spearman’s rho was used for the correlation analysis, specifically to measure the strength of a positive or negative association between two variables. The level of statistical significance was set at *p* < 0.05 (two-sided). The results are reported as median [IQ1–IQ3] or number of cases (%).

## 5. Conclusions

Further work is needed to determine whether the quantification of BNP and NT-BNP in LAA- and CE-derived AIS clots can serve as a predictive or prognostic biomarker or help to better understand the pathophysiology of AIS clots. From our observations, we can conclude that there is no significant difference in BNP and NT-proBNP expression in LAA and CE AIS clots. Therefore, we did not find any evidence that the brain natriuretic peptide levels in extracted clots can be used as specific biomarkers of cardioembolic stroke etiology, despite their utility as serum biomarkers of heart failure. We observed no correlation with the baseline clinical characteristics assessed. However, further work correlating BNP and NT-proBNP expression with longer-term patient outcome measures is needed. Nonetheless, as previously observed for other possible stroke biomarkers [48], our observation of the presence and localization of BNP and NT-proBNP in clots indicates that they might be involved in thrombosis, uncovering novel features of the thrombo-inflammatory pathway leading to pathological thrombus formation, which is certainly worth of further study.

## Figures and Tables

**Table 1:** Baseline clinical characteristics of the overall cohort of patients and of patients grouped according to stroke etiology.

	Overall Cohort of Patients (N = 80)	LAA (N = 40)	CE (N = 40)	Statistical Analysis
**Admission NIHSS ^a^**	14 [9–19]	15 [9–19]	14 [9–19]	H1 = 0.53, *p* = 0.818
**rtPA ^b^ administration**				
rtPA yes	39	20	19	*X*^1^ = 0.050, *p* = 0.823
rtPA no	41	20	21	
**Occluded vessel(s) ^c^**				
MCA, M1	41 (51.2%)	13 (32.5%)	28 (70.0%)	H1 = 16.460, *p* < 0.0001 *
MCA, M2	5 (6.3%)	2 (5.0%)	3 (7.5%)	
MCA, M3	1 (1.3%)	0 (0.0%)	1 (2.5%)	
MCA, not specified	2 (2.5)	1 (2.5%)	1 (2.5%)	
MCA (multiple branches/segments)	1 (1.3%)	0 (0.0%)	1 (2.5%)	
ICA and ICA terminus	6 (7.5%)	4 (10.0%)	2 (5.0%)	
ACA	1 (1.3%)	0 (0.0%)	1 (2.5%)	
VB	6 (7.5%)	4 (10.0%)	2 (5.0%)	
Tandem occlusion	15 (18.8%)	14 (35.0%)	1 (2.5%)	
3 Or more occluded vessels	2 (2.5%)	2 (5.0%)	0 (0.0%)	
**Final mTICI ^d^ score**				
mTICI 0	2 (2.5%)	1 (2.5%)	1 (2.5%)	H1 = 0.397, *p* = 0.529
mTICI 1	0 (0.0%)	0 (0.0%)	0 (0.0%)	
mTICI 2a	1 (1.3%)	1 (2.5%)	0 (0.0%)	
mTICI 2b	12 (15.0%)	7 (17.5%)	5 (12.5%)	
mTICI 2c	17 (21.3%)	8 (20.0%)	9 (22.5%)	
mTICI 3	48 (60.0%)	23 (57.5%)	25 (62.5%)	

^a^ NIHSS^:^ National Institutes of Health Stroke Scale, N = 77, 37 LAA and 40 CE patients. ^b^ rtPA: recombinant tissue plasminogen activator ^c^ MCA: middle cerebral artery; ICA: internal carotid artery; ACA: anterior cerebral artery; VB: vertebro-basilar. ^d^ mTICI: modified treatment in Cerebral Ischemia. Data shown as N (%) of cases or median [IQ1, IQ3]. * indicates a statistically significant difference.

## Data Availability

Data are contained within the article and Supplementary Materials.

## Supplementary Materials

The supporting information can be downloaded at: https://www.mdpi.com/article/10.3390/ijms25052999/s1.
