# Supplementary material for: Investigating the Role of Brain Natriuretic Peptide (BNP) and N-Terminal-proBNP in Thrombosis and Acute Ischemic Stroke Etiology
Source: Int J Mol Sci. 2024 Mar 5;25(5):2999. doi: 10.3390/ijms25052999 (PMC10931830; doi:10.3390/ijms25052999)

Supplementary Figure S1. IHC staining of positive and negative controls for the white blood cell markers: CD3 (**A**), CD66b (**B**), CD68 (**C**) and negative control (**D**) in human tonsil are provided.

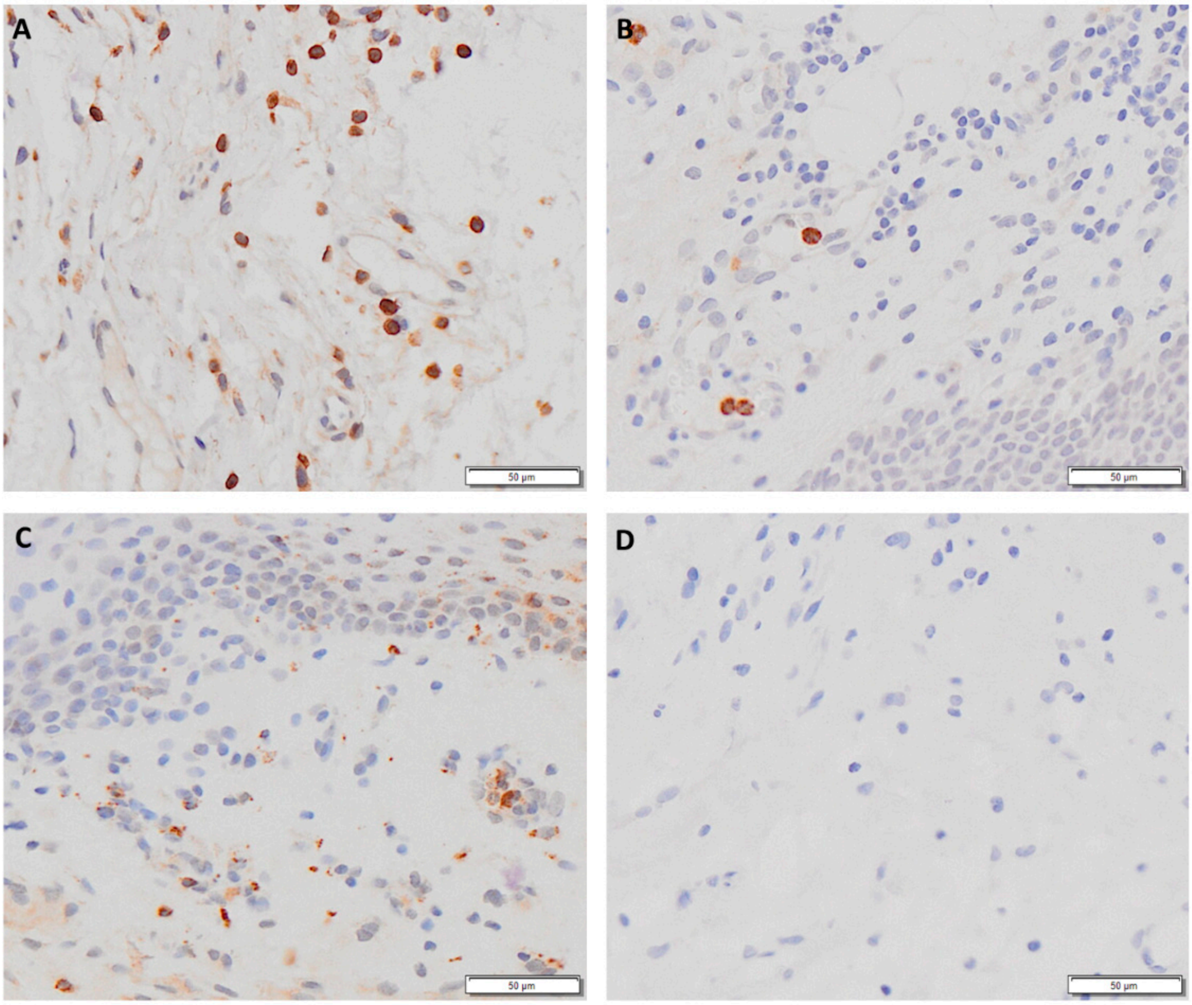

Supplement: Supplementary file 1 [file ijms-25-02999-s001.zip › ijms-2861155-supplementary.pdf]
